# Effect of the biopesticide Novoсhizol on the expression of defense genes during wheat infection with stem rust Puccinia graminis f. sp. tritici

**DOI:** 10.18699/vjgb-25-127

**Published:** 2025-12

**Authors:** A.B. Shcherban, A.V. Razuvaeva, E.S. Skolotneva, V.V. Fomenko

**Affiliations:** Institute of Cytology and Genetics of the Siberian Branch of the Russian Academy of Sciences, Novosibirsk, Russia; Institute of Cytology and Genetics of the Siberian Branch of the Russian Academy of Sciences, Novosibirsk, Russia; Institute of Cytology and Genetics of the Siberian Branch of the Russian Academy of Sciences, Novosibirsk, Russia; N.N. Vorozhtsov Novosibirsk Institute of Organic Chemistry of the Siberian Branch of the Russian Academy of Sciences, Novosibirsk, Russia

**Keywords:** biopesticide, chitosan, Novochizol, defense genes, stem rust, transcription, common wheat, биопестицид, хитозан, Новохизоль, гены защиты, стеблевая ржавчина, транскрипция, мягкая пшеница

## Abstract

Stem rust, caused by the fungus Puccinia graminis f. sp. tritici (Pgt), is a harmful disease affecting grain crops. The traditional way to combat this and other infectious plant diseases is to use chemical pesticides. Biopesticides, as well as plant disease resistance inducers – in particular those based on chitosan, a derivative of chitin – are increasingly being considered as an effective and safe alternative. Recently, a globular form of chitosan, Novochizol, has been developed, which has a number of advantages and has shown its effectiveness in preliminary field and laboratory experiments. However, there are no works devoted to the effect of this preparation on the expression of defense genes. Therefore, the aim of this work was to search for genes involved in the response of common wheat (Triticum aestivum L.) to stem rust infection and to evaluate the effect of Novochizol treatment on their transcription during the infection process. The wheat line ISr6-Ra with the stem rust resistance gene Sr6 and two Pgt isolates – an avirulent one, Avr6, and a virulent one, vr6 – were used as a model, allowing us to compare the effects of Novochizol depending on the genetic compatibility in the plant−pathogen pathosystem. To analyze the transcription level of defense genes, leaf material was collected at different time points from 3 to 144 h after inoculation of plants with the pathogen. Quantitative PCR analysis showed an increase in the transcription levels of the CERK1, PR3, PR4, PR5, PR6 and PR9 genes in plants treated with Novochizol and infected with various Pgt isolates compared to untreated infected plants. Pgt isolate Avr6 induced the highest expression of some defense genes (primarily CERK1), which is consistent with the phytopathology data showing the maximum degree of resistance (IT1) to stem rust in Novochizol-treated plants with a combination of Sr6–Avr6 genes. The data obtained confirm that one of the optimal strategies for increasing the resistance of grain crops to fungal pathogens is a combination of selection for specific resistance genes with the use of biological control agents.

## Abstract (ru)

Стеблевая ржавчина, вызываемая грибом Puccinia graminis f. sp. tritici (Pgt), является вредоносным заболеванием, поражающим посевы зерновых культур. Традиционный способ борьбы с этим и другими инфекционными болезнями растений − использование химических средств защиты. В качестве их эффективной и безопасной альтернативы все чаще рассматриваются биопестициды, а также индукторы болезнеустойчивости растений, в частности на основе хитозана, производного хитина. Недавно разработана глобулярная форма хитозана − Новохизоль, имеющая ряд преимуществ и показавшая свою эффективность в предварительных полевых и лабораторных экспериментах. Однако в настоящее время отсутствуют работы, посвященные влиянию данного препарата на экспрессию генов защиты. Поэтому целью данной работы стали поиск генов, принимающих участие в реакции растений мягкой пшеницы Triticum aestivum L. на заражение стеблевой ржавчиной, и оценка влияния обработки препаратом Новохизоль на их транскрипцию в ходе инфекционного процесса. В качестве модели были задействованы линия пшеницы с геном устойчивости к стеблевой ржавчине Sr6 и два отобранных изолята Pgt для этой линии: авирулентный (Avr6) и вирулентный (vr6), позволяющие сопоставить эффекты препарата Новохизоль в зависимости от генетической совместимости в патосистеме растение−патоген. Для анализа уровня транскрипции генов защиты использовали листовой материал в различных временных точках, от 3 до 144 ч после инокуляции растений патогеном. Количественный ПЦР-анализ показал повышение уровня транскрипции генов CERK1, PR3, PR4, PR5, PR6 и PR9 у растений, обработанных изучаемым биопестицидом и инфицированных различными изолятами Pgt, по сравнению с необработанными инфицированными растениями. Полученные данные подтверждают, что одна из оптимальных стратегий повышения устойчивости зерновых культур к грибным патогенам с точки зрения экологической безопасности – сочетание методов селекции по генам специфической устойчивости с применением биологических средств защиты.

## Introduction

Common wheat Triticum aestivum L. is the most popular and largest grain crop in terms of sown area. One of the main problems in its cultivation is the decrease in its yield due to diseases caused by various pathogens, especially fungi (Hafeez at al., 2021). Almost 90 % of the world’s wheat sown area is at risk of being affected by at least one fungal disease (Chai et al., 2022). Among the most dangerous are leaf-stem diseases that reduce the assimilation surface of leaves, and destroy chlorophyll, which leads to a decrease in photosynthesis, premature aging and death of plant tissues. Crop losses can range from 20 to 70 %. Often, such fungal infections take the form of epiphytoties. Since the beginning of 2000, the incidence of stem rust in wheat crops in Europe has increased sharply and continues to grow (Patpour et al., 2022). In the Russian Federation (RF), epiphytoties of stem and brown rust, septoria, and powdery mildew are periodically observed (Sanin, 2012).

In response to pathogen attacks, plants have developed a complex immune system that includes different levels of defense. The perception of pathogen-associated molecular patterns (PAMPs) by membrane pattern recognition receptors (PRRs) leads to PAMP-triggered immunity (PTI) (Bigeard et al., 2015). In an attempt to overcome this level of defense, the pathogen secretes specific avirulence effectors (Avr gene products) that suppress PTI, thus promoting further disease development. In response, plants have developed the next, specific level of defense based on intracellular recognition of effectors by NOD-like receptors (NLRs), products of plant R genes. This effector-triggered immunity (ETI) defense mechanism often results in programmed cell death in infected tissue or a hypersensitive response (HSR) that creates a barrier to pathogen dissemination (Jones, Dangl, 2006; Bent, Mackey, 2007).

PTI and ETI have been shown to be closely interrelated and jointly mediate systemic resistance to disease (Ngou et al., 2021). This resistance is based on the activation of a number of signaling cascades, including hormonal ones, which are controlled by hormones such as salicylic (SA), jasmonic (JA) and abscisic acids, ethylene, etc., depending on the type of nutrition of the pathogen: biotrophic, hemibiotrophic or necrotrophic (Jones, Dangl, 2006).

The salicylate-controlled cascade characteristic of the response against biotrophic pathogens, including the stem rust pathogen Puccinia graminis f. sp. tritici (Pgt), leads to the accumulation of reactive oxygen species (ROS) – an oxidative burst that provokes HSR, synthesis of phenolic substances and specific protective PR (pathogenesis-related) proteins (Tada et al., 2008; Ding et al., 2018).

PR proteins are the most important factors in plant resistance mechanisms. They participate in signaling systems, catalyzing the formation of hormones, strengthen the cell walls of plants, and are capable of causing damage to the cell walls and cytoplasmic membranes of pathogens (Tarchevskij, 2002). PR proteins are divided into 17 families (PR1−PR17) (Van Loon, Van Strien, 1999; Sels et al., 2008). Many of them are hydrolases. Thus, proteins of the PR2 family are β-1,3-endoglucanases; they accumulate around the site of infection, exhibiting fungicidal activity. Proteins of the PR3, PR4, PR8 and PR11 families have endochitinase activity and are capable of destroying the cell wall of fungi; their pool increases during infection. Representatives of the PR7, PR8, PR9 and PR10 families exhibit proteinase, lysozyme, peroxidase and ribonuclease activities, respectively. Proteins of the PR6 family are proteinase inhibitors that suppress proteolytic enzymes secreted by pathogens to penetrate plant tissues. The activity of other PR proteins is associated with an increase in membrane permeability (families PR5, PR12, PR13, PR14), as well as with the accumulation of hydrogen peroxide (families PR15 and PR16); the synthesis of these proteins is significantly increased by treatment with elicitors (Van Loon, Van Strien, 1999; Scherer, 2005; Van Loon et al., 2006).

One of the well-established long-term strategies for plant protection against pathogens is based on genetic protection using R genes, the inducers of ETI (see above). The gene catalog of the International Symposium on Wheat Genetics contains about 58 stem rust resistance genes (Sr) identified in wheat (McIntosh et al., 2011). Most of them, such as Sr25, Sr26, Sr31, Sr35, Sr38, Sr39, Sr44, Sr45, provide specific resistance mechanisms, including HSR and PR protein expression. However, the emergence of new pathogen races overcoming resistance in crop varieties increases losses from diseases. Therefore, the use of chemical pesticides became an additional line of defense, although it can have a negative impact on human health, food safety and the environment, and can also be toxic to non-target beneficial organisms. This stimulates the search for safe alternative plant protection products – biopesticides and resistance inducers (Ali et al., 2023).

Chitin is one of the most studied PAMPs that induce plant immunity. It is the main component of fungal cell walls, exoskeletons of arthropods, including insects. Chitosan, a biopesticide, was obtained by hydrolysis and deacetylation of chitin, the effect of which as a growth stimulator and inducer of non-specific resistance to fungal, bacterial and viral diseases has been confirmed in many studies (Maluin, Hussein, 2020; Shcherban, 2023). However, there are a number of disadvantages that complicate the use of chitosan: insolubility in water at physiological pH, low stability, variability of effects depending on the weight of the molecules, the degree of their deacetylation and the composition of the monomers (Katiyar et al., 2014; Varlamov et al., 2020).

Since there are currently no standard protocols for determining these parameters, assessments of the effects of chitosan preparations in different laboratories are often poorly comparable. Recently, scientists from the RF (patent US 20230096466A1; https://www.freepatentsonline.com/ y2023/0096466.html) obtained a globular chitosan form, Novochizol, which has a number of advantages: increased solubility in aqueous solutions, high adhesion to plant tissues, chemical stability, the ability to form complexes with other biologically active substances (Fomenko, Loroch, 2021). As a result of treating common wheat seeds with this preparation, their germination was accelerated, and an increase in root mass and total plant mass was observed (Teplyakova et al., 2022).

In field conditions, pre-sowing seed treatment and plant treatment with Novochizol in combination with natural fungicides had a positive effect on the growth and productive properties of wheat, and also increased its resistance to a number of fungal diseases (Orlova et al., 2025). At the same object, the inhibitory effect of Novochizol on the development of stem rust was demonstrated in laboratory conditions (Shcherban et al., 2024). It was shown that under the influence of treatment with this drug, ROS, in particular hydrogen peroxide (H2O2), accumulate in the leaf tissue of plants after infection with Pgt due to the modulation of the activity of antioxidant enzymes. It should be noted that the latter work studied the reaction of a variety with a complex genotype infected with a mixed population of the fungus (Shcherban et al., 2024).

It is of interest to study Novochizol-induced mechanisms of resistance to isolated Pgt races with contrasting virulence using a common wheat line carrying the corresponding Sr gene. For this purpose, we selected an isogenic line for the Sr6 gene, on which avirulent and virulent Pgt isolates were effectively differentiated (Skolotneva et al., 2020).

The aim of this work was to assess the susceptibility level of plants of this line treated with Novochizol and separately infected with one or another Pgt isolate, as well as to conduct a comparative analysis of the dynamics of expression of protection genes (PR) in the leaf tissue of plants within 144 hours after inoculation. To date, data have been accumulated on the effect of chitosan preparations on the expression of PR genes after plant infection with various pathogenic fungi (Manjunatha et al., 2008; Maluin, Hussein, 2020; Shcherban, 2023); however, in our work, the study of such an effect of the Novochizol preparation was carried out for the first time.

## Materials and methods

Plant material and stem rust pathogen. To obtain seed material, the common wheat line ISr6-Ra, kindly provided by Prof. J. Colmer (St. Paul, USA), was grown hydroponically in the artificial climate laboratory of the Institute of Cytology and Genetics SB RAS. To confirm the presence of the stem rust resistance gene Sr6 in this line, the plant material was genotyped using the molecular marker Xcfd43 to this gene (Tsilo et al., 2009). In the laboratory experiment, plants were grown in vessels with soil, under conditions recommended for work with rust fungi by international protocols (Roelfs et al., 1992). For treatment with Novochizol, 10-day-old seedlings were used, and as a control, uninfected plants without treatment, grown in parallel with the experimental samples, were used.

Previously, the West Siberian population of Pgt was genotyped for races of the fungus containing different combinations (pathotypes) of Avr and vr genes (Skolotneva et al., 2020). In our experiment, we used the LKCSF (vr6)/ LCCSF (Avr6) pathotype system with the highest yield of urediniospores. The selected isolates were propagated on the susceptible variety of common wheat Khakasskaya to obtain spore material in the amount required to infect an experimental batch of plants at the rate of 10 mg per 1,000 plants. The spore material was stored at a temperature of −70 °C until the experiment.

Novochizol treatment and Pgt infection. Assessment of plant susceptibility. Novochizol with a deacetylation degree of ≥90 % and a molecular weight of ~500 kDa was provided by Novochizol SA (Monte, Switzerland). Aqueous suspensions of this preparation were prepared as described previously (Teplyakova et al., 2022). A concentration of 0.125 %, which had previously been shown to be effective against stem rust (Shcherban et al., 2024), was chosen for the treatment of 10-day-old plant seedlings. Novochizol suspension was applied to plants using a household sprayer (15 ml/100 plants) four days before Pgt inoculation.

For plant inoculation, Pgt urediniospores were premixed with Novec 7100 mineral oil (3M Novec™, St. Paul, MN, USA) at a concentration of 25 mg spores per 5 ml oil and applied to seedlings using a sprayer. Control plants were treated with bidistilled water. Inoculated plants were incubated for 24 h in a humidified chamber, in the dark, at 15–20 °C to ensure maximum spore germination. The plants were then transferred to growth chambers and incubated for 16 h under 10,000 lux phytolamp illumination and at a temperature of 26–28 °C. These conditions are necessary for the formation of appressoria, pathogen penetration into the stomata, and the development of infectious hyphae in the intercellular spaces of the plant (Roelfs et al., 1992).

The degree of plant susceptibility to stem rust was assessed taking into account the qualitative (type of reaction) and quantitative components of this indicator. Infection types of plant reaction (IT) were determined 12–14 days after inoculation using the modified Steckman scale (Roelfs et al., 1992), where IT “0”, “;”, “1” and “2” were interpreted as resistant (Low, L), and “3”, “3+” and “4”, as susceptible (High, H).

RNA extraction, reverse transcription, RT-PCR and qPCR. For sample collection at different time points after infection (3, 6, 24, 48, 72, 144 h), three plants were planted as biological replicates for each experimental sample. Individual leaves were cut from each plant and immediately placed in liquid nitrogen. The collected material was stored at –80 °C before RNA extraction. Total RNA was extracted using the R-PLANTS-kit (Biolabmix, Novosibirsk, Russia) according to the manufacturer’s instructions. Genomic DNA was removed using heat-labile DNase (Biolabmix). Reverse transcription was performed using the OT-M-MuLV-RH first-strand cDNA synthesis kit (Biolabmix) using 1 μg of total RNA and oligo(dT)16 as a primer.

For reverse transcription-PCR (RT-PCR), the conditions were the same as for qPCR (see below). RT-PCR products were analyzed by electrophoresis in 1.5 % agarose gel followed by staining with ethidium bromide.

The BioMaster HS-qPCR Lo-ROX SYBR (2×) reagent kit (Biolabmix) and the QuantStudio 5 Real-Time PCR System (Thermo Scientific, USA) were used for qPCR. The sequences of gene-specific primers are listed in the Table. Amplification conditions were 5 min at 95 °C, followed by 40 cycles of 10 s at 95 °C, 20 s at 60 °C (55 °C for the GAPDH gene), and 10 s at 72 °C. qPCR data were collected during each round of amplification. Melting curve analysis between 60 and 95 °C was applied to all products to determine the fidelity of the PCR reaction.

**Table 1.**
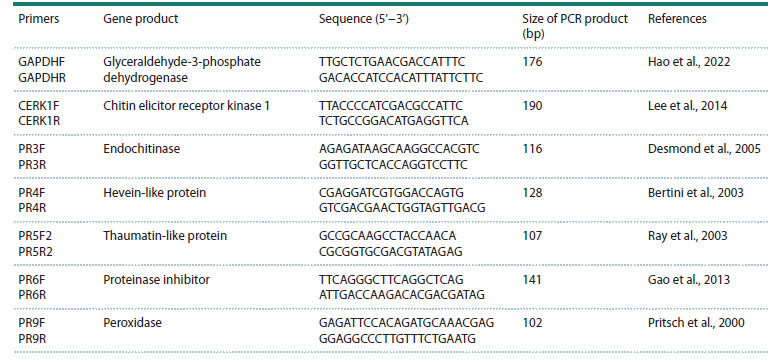
Primer sequences and expected sizes of PCR products

Gene expression measurements were performed in three biological replicates, each with three technical replicates. Relative mRNA amounts were determined using Pfaffl’s formula (Pfaffl, 2001). PCR efficiency was assessed using LinReg software (Ruijter et al., 2009). mRNA expression levels were normalized to the level of the housekeeping gene GAPDH. This gene was chosen as a reference because no differences in its mRNA content were detected between Pgt-infected and control samples (data not shown). Analysis of variance with Tukey’s post hoc test was used to compare expression levels.

To assess the transcription level of common wheat genes induced during Pgt infection using qPCR, we selected six genes for which preliminary RT-PCR revealed the presence of a monomorphic amplification product of the expected length with varying intensity at individual time points after inoculation with the pathogen (see the Table).

## Results

**Effect of Novochizol on stem rust development**

To study the effect of Novochizol treatment on the susceptibility of common wheat plants to stem rust and the expression of defense genes, we used a model including the T. aestivum ISr6-Ra line and the corresponding Avr6 and vr6 Pgt isolates. This model allowed us to study various resistance mechanisms depending on the recognition of pathogen ligands by the host’s immune receptors.

**Fig. 1.**
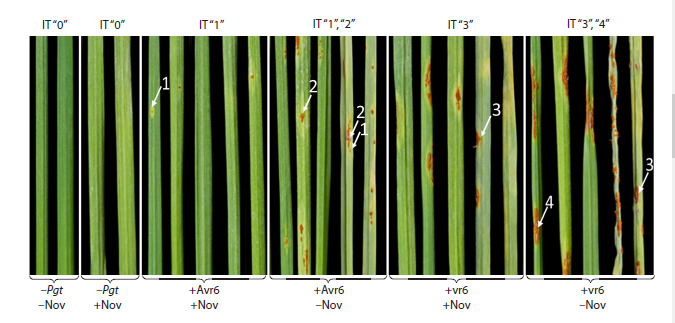
Effect of Novochizol on the development of stem rust symptoms on leaves of wheat of the T. aestivum ISr6-Ra line. Avr6 and vr6 are avirulent and virulent isolates of the stem rust pathogen (Pgt). Infectious types of plant response (IT) are indicated above the figure. Pustules characteristic of each IT are indicated by arrows. Nov – Novochizol. Photofixation was carried out on the 14th day after inoculation with the pathogen. The age of plants at the time of fixation was 28 days.

Fourteen days after inoculation, the susceptibility level of plants infected with the Avr6 isolate corresponded to the resistant type (IT = 1−2) (Fig. 1). This scenario corresponds to cases of interaction of plant/pathogen genotypes carrying complementary genes of resistance and avirulence, i. e. encoding the immune receptor and its specific ligand. Infection with the vr6 isolate resulted in a susceptibility reaction (IT = 3−4). An earlier assessment of the quantitative component of resistance, determined by the average number of pustules per leaf blade, showed a multifold decrease in the number of pustules in plants pretreated with Novochizol compared to untreated plants, in the case of both isolates (data not published). As a result of treatment, plants infected with Avr6 and vr6 were similar in terms of the quantitative indicator (~1.5 pustules per leaf on average); however, in general, they retained the specific IT of resistance and susceptibility for each isolate, determined by the size of the pustules (Fig. 1).

**Transcription level assessment of defense genes**

Induction of the CERK1 gene during Pgt infection of wheat. Chitin elicitor receptor kinase 1 (CERK1) has been identified as a PAMP receptor for chitin, a major structural component of fungal cell walls (Gong et al., 2020; Wang L. et al., 2024). Upon recognition of chitin, this receptor protein activates various defense-related signaling pathways, playing a key role in plant immunity

In untreated plants infected with the vr6 isolate, a slight increase in transcription of this gene was detected at 24 hours post inoculation (h/i), while at other points the transcription level did not exceed the control. In the group of untreated plants infected with the Avr6 isolate, no increase in the amount of CERK1 transcripts was observed throughout the experiment, compared to the control.

**Fig. 2.**
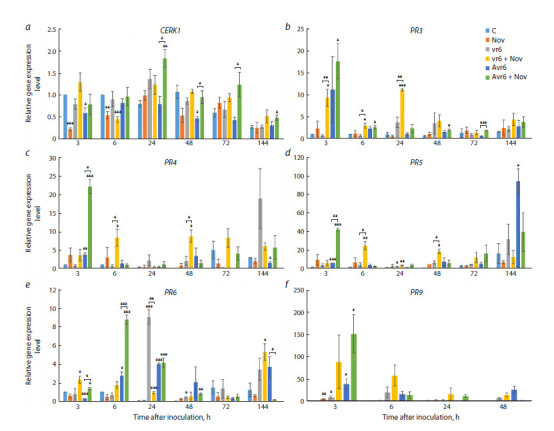
Dynamics of gene transcription of some protective proteins in leaves of common wheat line ISr6-Ra after their treatment with 0.125 % Novochizol and inoculation with the vr6 or Avr6 − Pgt isolate C – control; Nov – treatment with the preparation; vr6 – inoculation with isolate vr6; vr6 + Nov – treatment with the preparation and inoculation with isolate vr6; Avr6 – inoculation with isolate Avr6; Avr6 + Nov – treatment with the preparation and inoculation with isolate Avr6. Columns represent the average level of gene transcripts relative to the reference (± standard error of the mean). Comparisons were made with control plants at individual time points and pairwise comparisons of vr6/vr6 + Nov and Avr6/Avr6 + Nov (in parentheses); p-values: * p ≤ 0.05, ** p ≤ 0.01, *** p ≤ 0.001.

Novochizol-pretreated plants showed different responses to Pgt infection depending on the isolate type (Fig. 2a). In vr6-infected plants, we observed an increase in CERK1 transcript levels at 3 and 48−144 h/i relative to untreated plants infected with the same isolate, although the significance of this increase at individual points was low. At the same time, Avr6-infected plants pretreated with the drug showed a significant increase in CERK1 transcription by approximately 2−3 times relative to untreated plants in the range of 24−72 h/i. In uninfected plants treated with the preparation, the level of CERK1 transcription did not significantly exceed that of the control plants, and at some points even decreased (Fig. 2a). Thus, although CERK1 induction under the influence of the biopesticide Novochizol was observed for both isolates, the degree of this induction was higher in the case of the Avr6 isolate.

Induction of the PR3 and PR4 genes encoding various chitinases. The PR3 and PR4 genes encode proteins with chitinase activity that cleave the chitin of pathogenic fungi, herbivorous insects, and nematodes (Van Loon et al., 2006). These enzymes do not have an endogenous substrate and play an important role in the lysis of the cell walls of many pathogens

Transcription of both genes in control plants and plants treated with Novochizol without infection was approximately at the same level, without any significant changes (Fig. 2b, c). In untreated plants infected with vr6, a slight increase in the PR3 transcription level was observed at 24 h/i, followed by a decrease to the initial level on the third day after inoculation. In untreated Avr6-infected plants, an elevated mean PR3 transcription level was observed at 3 h/i; no significant changes were observed relative to controls at other time points. Regarding PR4, the expression of this gene in untreated vr6-infected plants showed an increase in the mean level at 144 h/i, whereas induction was observed as early as 3 h/i after Avr6 infection.

Treatment with the preparation induced higher levels of PR3 and PR4 gene transcription in response to Pgt infection compared to untreated plants. Thus, in the case of vr6 infection of treated plants, we observed increased transcription of the PR3 gene after 3–24 h/i with a maximum level at 24 h/i, and for PR4, after 6 and 48–72 h/i. Transcription of both genes increased after 3 h/i when plants were infected with the Avr6 isolate, and the statistically most significant increase compared to untreated plants was found for the PR4 gene (Fig. 2b, c).

Expression of PR5, PR6 and PR9 genes. In this work, we also studied the dynamics of transcription of the PR5, PR6 and PR9 genes, which play an important role in plant defense against pathogens. The PR5 gene encodes a thaumatin-like protein with antifungal activity and is also an osmoregulator that protects plants from abiotic stress (Van Loon et al., 2006). The PR6 gene product is a proteinase inhibitor that suppresses the pathogen’s proteolytic enzymes. The peroxidase encoded by the PR9 gene plays an important role in strengthening the plant cell wall and in the formation of ROS to create a toxic environment for the pathogen (Almagro et al., 2009).

The transcription levels of the PR5, PR6 and PR9 genes in the control and uninfected plants treated with Novochizol did not undergo significant changes during the experiment (Fig. 2d–f). In untreated plants infected with one or another Pgt isolate, accumulation of PR5 transcripts was observed only on the 6th day of infection with the pathogen (Fig. 2d). Under the influence of the preparation, an earlier increase in the PR5 transcription level was observed in plants infected with vr6, especially noticeable after 6 and 48 h/i. In plants infected with Avr6, pretreatment with the preparation caused a significant induction of PR5 transcription after 3 h/i with a subsequent decrease in the expression level. It should be noted that there are signs of similarity in the dynamics of PR5 transcription with that of the PR3 and PR4 genes (see above).

In untreated vr6-infected plants, PR6 transcription peaked at 24 h/i, whereas in untreated Avr6-infected plants, transcription of this gene increased less significantly at 6 and 24 h/i (Fig. 2e). Interestingly, biopesticide treatment caused a significant decrease in PR6 transcript levels in vr6-infected plants at 24 h/i. In the case of infection with Avr6, maximum PR6 expression in treated plants, significantly higher than in untreated plants, was observed at 6 h/i, and then the levels of both groups of plants became similar except at 144 h/i.

In untreated vr6 infected plants, PR9 transcript levels increased between 3 and 6 h/i and then decreased (Fig. 2f). In treated vr6 infected plants, PR9 transcript levels increased between 3 and 24 h/i with high interplant variability. A lesser increase in expression of this gene was observed in treated Avr6 infected plants, except for 3 h/i, which was the time point at which the highest PR9 transcript level was recorded. PR9 transcripts were not detected at 72 and 144 h/i in control and infected samples

## Discussion

The effect of chitosan preparations on defense reactions and signaling pathways that control immunity has been studied in detail in various plant objects, although not enough to fully understand the mechanisms of this effect (review: Shcherban, 2023). However, so far there have been only a few studies devoted to the analysis of the effects of Novochizol, a globular derivative of chitosan (Teplyakova et al., 2022; Shcherban et al., 2024; Orlova et al., 2025). Our work provides new information on the changes in the expression of defense genes induced by this preparation during infection of wheat with the causative agent of a dangerous fungal disease, stem rust.

In response to pathogen attacks, plants have developed a complex immune system, including PRRs, PTI-mediated receptors that represent the first line of plant defense against infections. PRRs are central elements of plant immunity, triggering a cascade of defense reactions, including ROS accumulation, PR gene expression, and other responses (Jones and Dangl, 2006).In our study, we analyzed the effect of Novochizol on the expression of the gene encoding PRR CERK1 in leaf tissue of wheat plants infected with different Pgt isolates. In response to infection with avirulent and virulent isolates of Pgt, plants not treated with the preparation did not show significant changes in the expression level of this gene throughout the experiment (Fig. 2a). Also, no significant changes in the expression level were detected in uninfected plants treated with the preparation.

Previously, activation of CERK1 gene transcription in ear tissue was shown under the influence of chitin and during infection with the causative agent of fusarium Fusarium graminearum Schwabe (Wang L. et al., 2024). Apparently, the causative agents of Fusarium and stem rust provoke different reactions of the defense system in various plant tissues, and chitin, a natural elicitor, can provoke a more pronounced reaction compared to the Novochizol preparation.Plants treated with the preparation demonstrated a more pronounced response to infection with Pgt, which depended on the isolate type. So, when plants were infected with vr6, we observed a slight increase in the CERK1 transcription level after 3 h/i and in the range of 48–144 h/i, whereas when plants were infected with the avirulent isolate Avr6, the degree of CERK1 induction was higher than in the previous case and exceeded the expression level of this gene in untreated plants by 2–3 times in the range of 24–72 h/i. This increase in Avr6-infected plants can be explained by the close relationship between PTI and ETI.

Previous studies have shown that these levels of defense, activated in plants by surface and intracellular receptors, respectively, are mutually reinforcing (Ngou et al., 2021). From this perspective, it can be speculated that the presence of an intracellular plant receptor (Sr6 gene product) complementary to the Avr6 product of the Pgt gene is an additional trigger capable of enhancing PTI by increasing CERK1 gene expression. Recently, CERK1 overexpression has been shown to confer broad-spectrum resistance to fungal diseases in wheat (Wang L. et al., 2024).

Genes encoding the PR3 and PR4 proteins have endochitinase activity, are capable of destroying the cell wall of fungi, and are activated when plants are exposed to various pathogens (Van der Bulcke et al., 1990; Ward et al., 1991; Hammond-Kosack, Gones, 1996; Gao et al., 2013). According to the data obtained in the work, upon infection with vr6, the level of PR3 gene transcription increased after 24 h/i, while the PR4 gene was induced much later, on the 6th day after inoculation (Fig. 2b, c). During Avr6 infection, a synchronous increase in the expression of both genes occurred at 3 h/i

As in the case of CERK1, treatment with the drug caused an increase in the transcription of both chitinase genes, compared to untreated plants, which was most pronounced in the case of the Avr6 isolate at the same point as in untreated Avr6-infected plants – 3 h/i. Thus, the PR3 and PR4 genes are characterized by early accumulation of transcripts during infection with the Avr6 isolate, while Novochizol has an additional stimulating effect on the expression of these genes. It has been previously shown that treatment with chitosan preparations causes early accumulation of chitinases, which non-specifically destroy the chitin of fungal cell walls and thereby increase plant resistance to a wide range of fungal diseases (Manjunatha et al., 2008; Ma et al., 2013; Elsharkawy et al., 2022).

Numerous studies have shown that overexpression of thaumatin-like proteins (TLPs) belonging to the PR5 family provides significant plant resistance to various pathogenic fungi (Wang X. et al., 2010; Cui et al., 2021). We showed that, similar to the PR4 gene, the accumulation of PR5 transcripts occurred late – at 144 h/i in untreated plants infected with vr6 (Fig. 2d). Treatment with the drug resulted in earlier accumulation of PR5 transcripts at 6 and 48 h/i in plants infected with the same isolate. Finally, similar to PR4, PR5 showed significant induction of its expression at 3 h/i in treated plants infected with Avr6. Such similarity in the dynamics of PR4 and PR5 expression indicates a common mechanism of regulation of these genes, depending on the pathogen genotype.

Regarding the possible target of PR5 protein action, it was shown that overexpression of TLP in grape Vitis vinifera L. leads to a decrease in hyphal growth of the pathogenic fungus Plasmopara viticola (He et al., 2017). The exact mechanism of TLP action has not been established, however, PR5 was reported to degrade β-1,3-glucans, which, like chitin, are an important structural component of fungal cell walls (Grenier et al., 1999).

An important role of the PR6 gene product is to inhibit pathogen proteinases that degrade plant proteins (Ryan, 2000). According to our data, the expression of this gene in untreated plants showed a pattern similar to that of other PR genes studied in this work: a slight increase in the transcription level at 24 h/i in the case of vr6 and at 6, 24 and 144 h/i in the case of Avr6 (Fig. 2e). A special feature of this gene is its different response to Novochizol, depending on the type of isolate, at 24 h/i. The vr6 isolate at this point showed a sharp decrease in PR6 transcription as a result of treatment, whereas for Avr6, the transcription levels in treated and untreated plants were approximately the same. In the previous 6 h/i, we observed a more typical picture for Avr6, namely a significant increase in PR6 transcription in treated plants compared to untreated ones. It can be assumed that the peak of PR6 functional activity occurs in the period up to 24 h/i, after which its role decreases, which causes variability in the transcription level.

The PR9 gene encodes peroxidase, which is involved in hydrogen peroxide detoxification and lignin synthesis. The latter substance, accumulating in the plant cell wall, creates a barrier to pathogen spread (Andreeva, 1988). In vr6-treated infected plants, we observed an increase in PR9 transcription after 3–24 h/i (Fig. 2f). Treated plants infected with Avr6 showed an increased average transcription level after 3 h/i and no increase at other time points relative to untreated plants infected with the same isolate. The increased dispersion of data observed for both isolates can be explained by the uneven distribution of pustules on individual leaves (Fig. 1); the highest level of peroxidase activity and, accordingly, transcription of this gene can be expected in the areas of formation of morphological structures of the fungus (see below).

Nevertheless, these data correlate with a recent biochemical analysis, which showed an increase in the enzymatic activity of peroxidase in the 6–144 h/i period in Novochizol vr6-infected plants (data not published). We assume that the increase in peroxidase activity in this group of plants under the influence of the preparation is associated with the acceleration of the lignification process. Previous histochemical analysis revealed earlier and more intensive accumulation of phenolic substances in the cytoplasm and lignin on the cell walls in the Pgt colony zones in plants treated with the preparation compared to untreated plants (Shcherban et al., 2025).Thus, the induction of defense genes in plants treated with Novochizol is enhanced in almost all cases after pathogen inoculation, which is consistent with the previously proposed hypothesis of priming, a rapid response of the immune system to a subsequent pathogen attack under the influence of elicitors (Conrath, 2011).

## Conclusion

The data obtained indicate that the biopreparation Novochizol increases the quantitative component of resistance of the isogenic line ISr6-Ra to stem rust both in the case of the Avr6 isolate Pgt and in the case of the vr6 isolate. Analysis of the transcription of the defense genes CERK1, PR3, PR4, PR5, PR6 showed an increase in the transcription level of these genes in plants treated with the preparation and infected with various isolates of Pgt compared to untreated infected plants. The data obtained indicate that the use of the biopesticide Novochizol in combination with selection for resistance genes (pyramiding) is an effective way to increase the resistance of common wheat to stem rust.

## Conflict of interest

The authors declare no conflict of interest.
